# 

**Published:** 1884-12

**Authors:** 


					﻿DECEMBER, 1884.
THIRTY-FIRST YEAR.

JOUB1UL
OF

E. H. GIBBS, A.M., M.D., Editors
No. 75 & 77 BARCLAY STREET NEW YORK.
TERMS: $1 a Year.
SiDgle number, 10 cts.1
				

